# 

**Published:** 1904-07

**Authors:** 


ESTABLISHED IN 1839

THE OLDEST DENTAL MAGAZINE IN THE WORLD
INDEPENDENT---------PUBLISHED BY DENTISTS FOR DENTISTS

VOLUME XXXV
NUMBER 7

THIRD SERIES

JULY
1904

EDITOR:
WM. GIRD BEECROFT, D. D. S., Madison, Wis.
ASSOCIATE EDITORS:
ALBERT H. KETCHAM, D. D. S., Denver, Colo.
EMORY A. BRYANT, D. D. S., Washington, D. C.
GEO. S MARTIN, D. D. S., L. D. S., Toronto Junction, Ontario.

Publisheu on the 10th day of each month.
Subscription Price, $1.00 per year.
Foreign Countries, $1.50 pe: year.

Address all communications, both business and editorial to the editor.

**Entered as Second Class Matter, Madison, Wis.**

### THE FOURTH INTERNATIONAL DENTAL CONGRESS BANQUET

Will be held on September, the first, nineteen hundred and four, at eight P. M., in the Coliseum, adjoining the Congress Hall. The price per plate is three dollars. It is requested that all who expect to attend send their names and money to Dr. A. H. Fuller, P. O. Lock-box 604, St. Louis, Missouri, at once and not later than August the twentieth. Arrangements to pay can be made with Dr. A. H. Fuller at the time of registration, provided notice is given before August the twentieth.

G. A. Bowman,
A. H. Fuller,
Adam Flickinger,
*Banquet Committee.*

